# Proteome changes underpin improved meat quality and yield of chickens (*Gallus gallus*) fed the probiotic *Enterococcus faecium*

**DOI:** 10.1186/1471-2164-15-1167

**Published:** 2014-12-23

**Authors:** Aijuan Zheng, Jianjie Luo, Kun Meng, Jianke Li, Shu Zhang, Ke Li, Guohua Liu, Huiyi Cai, Wayne L Bryden, Bin Yao

**Affiliations:** Key Laboratory of Feed Biotechnology of Ministry of Agriculture, Feed Research Institute, Chinese Academy of Agricultural Sciences, Beijing, 100081 P. R. China; Key Laboratory of Pollinating Insect Biology of Ministry of Agriculture, Institute of Apicultural Research, Chinese Academy of Agricultural Sciences, Beijing, 100081 P. R. China; School of Agriculture and Food Sciences, University of Queensland, Gatton, QLD 4343 Australia

**Keywords:** Broiler chicken (*Gallus gallus*), *Enterococcus faecium*, Carcass, Meat quality, Pectoral muscle proteome, Probiotics

## Abstract

**Background:**

Supplementation of broiler chicken diets with probiotics may improve carcass characteristics and meat quality. However, the underlying molecular mechanism remains unclear. In the present study, 2D-DIGE-based proteomics was employed to investigate the proteome changes associated with improved carcass traits and meat quality of Arbor Acres broilers (*Gallus gallus*) fed the probiotic *Enterococcus faecium*.

**Results:**

The probiotic significantly increased meat colour, water holding capacity and pH of pectoral muscle but decreased abdominal fat content. These meat quality changes were related to the altered abundance of 22 proteins in the pectoral muscle following *E. faecium* feeding. Of these, 17 proteins have central roles in regulating meat quality due to their biological interaction network. Altered cytoskeletal and chaperon protein expression also contribute to improved water holding capacity and colour of meat, which suggests that upregulation of chaperon proteins maintains cell integrity and prevents moisture loss by enhancing folding and recovery of the membrane and cytoskeletal proteins. The down-regulation of β-enolase and pyruvate kinase muscle isozymes suggests roles in increasing the pH of meat by decreasing the production of lactic acid. The validity of the proteomics results was further confirmed by qPCR.

**Conclusions:**

This study reveals that improved meat quality of broilers fed probiotics is triggered by proteome alterations (especially the glycolytic proteins), and provides a new insight into the mechanism by which probiotics improve poultry production.

**Electronic supplementary material:**

The online version of this article (doi:10.1186/1471-2164-15-1167) contains supplementary material, which is available to authorized users.

## Background

Poultry is a significant source of animal protein and accounts for 30% of global meat consumption [1]. Compared with beef and pork, chicken meat contains lower concentrations of fat, sodium and cholesterol and a high degree of unsaturated fatty acids and a balanced *n*-6 to *n*-3 polyunsaturated fatty acid ratio [2, 3]. With the application of modern breeding technology, the major biological characteristics of meat chickens or broilers, i.e. pectoral (breast) muscle yield, body composition, daily weight gain, feed conversion efficiency and resistance to disease, have been improved significantly [4, 5]. Despite the success of breeding programs in increasing meat production, the high selection intensity has resulted in negative impacts on meat quality [6], including meat pH, water holding capacity, texture, and colour [7–9]. Moreover, meat flavour, storage and processing quality have also decreased following selection for increased meat production [10, 11]. As these negative effects on meat quality will impact on consumer acceptability of poultry meat [12], the physicochemical and sensory properties of broiler meat are receiving considerable research attention [13]. Water loss can reduce the nutritional value, flavor and tenderness of meat [14]. Moreover, pH is negatively correlated with water loss from muscle [15, 16]. Thus, pH, colour and water holding capacity of meat including drip loss and cooking loss are important parameters related to meat quality.

With reduced use of feed antibiotic growth promoters to satisfy consumers’ requirement, farmers employ several strategies to maintain chicken health and improve meat quality [17–19]. Dietary supplementation with probiotics is becoming popular. The live probiotic microbes can improve the intestinal environment by inhibiting pathogens through competition for nutrients and binding sites on the intestinal epithelium, promoting antimicrobial conditions, and stimulating the immune system [20]. Positive effects of numerous probiotics organisms (*Lactobacillus* spp., *Enterococcus faecium*, *Bifidobacterium bifidum*, *Bacillus subtilis*, *Streptococcus thermophdus*, *Pediococcus pentosaceus* or *Saccharomyces cerevisiae*) have been reported in chickens [21–31], while a few studies have shown no improvement in bird performance with dietary addition of probiotics [32]. Importantly for consumers, dietary supplementation of broiler diets with probiotics can improve carcass characteristics [33] and meat quality [34]. However, the molecular details of how probiotics improve the meat quality remain unclear. This is a complex area as meat quality is influenced by a wide spectrum of factors, including genetics, nutrition, husbandry conditions and handling before and after slaughter [35].

Proteomics has emerged as an effective approach for delineating the molecular basis of the physiological changes in muscle during chicken growth [36]. This approach should also assist in elucidating the mechanism of probiotic action [37, 38]. In this study, proteome changes were determined in the pectoral muscle of broiler chickens fed a probiotic to gain a better understanding of the mechanisms underlying improved carcass characteristics and meat quality induced by feeding probiotics to meat chickens.

## Methods

The study described in this paper was conducted in the Feed Research Institute, Chinese Academy of Agricultural Sciences (CAAS), Beijing, China. The care and use of all experimental birds was approved by the Animal Care and Use Committee of the Feed Research Institute of the CAAS.

### Materials and reagents

Microcapsules of *E. faecium* CGMCC 2516 [39, 40] (viable count ≥1 × 10^10^ cfu/g; Challenge Biotechnology Ltd. Co., Beijing, China) were used in the present experiment. All reagents for 2-D DIGE were purchased from GE Healthcare (Uppsala, Sweden), Bio-Rad (Hercules, CA, USA), Roche (Mannheim, Germany), and Sigma-Aldrich (St. Louis, MO, USA). The reagents for LC-Chip electrospray ionization quadrupole time-of-flight mass spectrometry (ESI-QTOF-MS) were purchased from Bruker Daltonics (Billerica, MA, USA), Roche, and J. T. Baker (Phillipsburg, NJ, USA).

### Bird management and experimental treatments

A total of 144 one-day-old, male, Arbor Acres (AA) broiler chickens that had been vaccinated with infectious bronchitis, avian influenza, Marek’s disease and Newcastle disease vaccines were purchased from Huadu Chicken Co. (Beijing, China). The chicks were randomly divided into two groups (control and treatment). Each group had 6 replicates (cages) and each replicate (cage) had 12 birds. The distribution of cages was arranged to avoid any location effects within the poultry house. The chickens were reared from 0 to 42 days and fed corn-soybean meal starter (0–21 days) and grower (22–42 days) diets (Additional file 1: Table S1) without (control) or with the probiotic (>10^6^ cfu/g *E. faecium*) for 42 days. All chickens were subject to a photoperiod of 23 h light and 1 h dark on days 0–7, and a photoperiod of 20 h light and 4 h dark thereafter in accordance with the AA Broiler Management Guide [41]. The room temperature was maintained at 33–35°C on days 0–3, at 32–34°C on days 4–7 and gradually reduced to the maintenance temperature of 20°C by day 42. The relative humidity was kept at 70% during the first week and thereafter at about 60%.

### Sample collection and parameter determination of carcass and meat traits

Three birds of each replicate (cage) of each group (n = 3 × 6 × 2) were selected randomly, weighed, electrically stunned, and manually slaughtered within 5 min [42]. The muscle of the middle part of left pectoralis was sampled and washed with PBS buffer (NaCl 8 g/L, Na_2_HPO_4_ 1.44 g/L, KH_2_PO_4_ 0.24 g/L, KCl 0.2 g/L, pH 7.2) to remove any blood and surface contaminants and immediately froze in liquid nitrogen and stored at −80°C for molecular analysis including 2DIGE and qPCR validation.

The carcass traits of chicken fed *E. faecium* or not were evaluated. Another three birds were randomly selected from each replicate (cage) of each group (n = 3 × 6 × 2). The percentage by weight of pectoral (breast) muscle, leg muscle (drumstick) and abdominal fat to live body weight was each determined. The entire pectoralis of birds were collected; the left for the measurement of water-holding capacity and the right for pH and meat colour determination. Water-holding capacity was determined as drip loss and weight loss after cooking [43]. In drip loss determination, approximately 30 g of regular-shaped muscle (denoted as W_45 min_) was hung in an inverted dixie cup within a zip-sealed plastic bag that was then filled with nitrogen to avoid oxidation, evaporation, and mutual extrusion. All bags were stored at 4°C for 24 h and the surface moisture of the fillets was then absorbed with filter paper and the fillets reweighed (W_24 h_) [44, 45]. Drip loss was calculated by the following equation:


Cooking loss was measured at 72 h post mortem as follows: about 20 g of regular-shaped muscle was removed from refrigerator (4°C), and the surface moisture of the fillets was absorbed with filter paper. After 20-min heating in a zip-sealed plastic bag in a water bath at 80°C, the meat was allowed to cool to ambient temperature, blotted dry and reweighed. The cooking loss was calculated as the percent weight loss [46].

The pH values of the pectoral muscle were measured at 45 min and 24 h post mortem at a depth of 2-cm using a portable pH meter (Testo 205, Testo AG, Lenzkirch, Germany) [47]. The pH meter was calibrated using buffer solutions (pH = 4.0 and pH = 7.0) after every 50 observations [48].

The colour of the fleshiest part near the top of the right pectoral muscle was assessed at 45 min and 24 h post mortem with a spectrophotocolourimeter (Minolta CR-400, Konica Minolta Sensing, Osaka, Japan) using the CIELAB system. The instrument was calibrated with a white-and-black tile before analysis in agreement with the International Commission on Illumination [49].

All data were presented as means ± SD (n = 18) and subjected to a one-way ANOVA procedure provided in SPSS16.0 software. Treatment difference was assumed to be statistically significant when *p* ≤ 0.05 unless otherwise stated.

### Two-dimensional fluorescence difference gel electrophoresis (2-D DIGE)

Total protein extraction of muscle was carried out as described previously with some modifications [50]. The pectoral muscle samples of three chicken from each replicate (cage) were combined as a biological replicate (n = 6 × 2), homogenized by pestle in liquid nitrogen and dissolved in 1 mL of PBS buffer (pH 7.0) containing EDTA-free protease inhibitor cocktail tablets (Roche). The proteins, insoluble in PBS, were extracted by lysis buffer (9 M urea, 2 M thiourea, 4% CHAPS and EDTA-free protease inhibitor cocktail tablets, pH 8.5) and combined with the PBS soluble proteins. Trichloroacetic acid was added at the ratio of 1:9, followed by 10-min incubation at −20°C. After centrifugation at 15,000 × *g* and 4°C for 10 min, the pellet was washed with cold acetone, incubated and re-centrifuged as described above. The pellet was washed three times, air dried, suspended in lysis buffer at the ratio of 1 mg:10 μL, and sonicated for 2 min. The protein concentration of the supernatant was determined by the 2-D Quant Kit (GE Healthcare).

Three 2-D DIGE gels (technical replicates) of each biological replicate were run as described by Lu et al. [51] with some modifications. The pH of the protein solutions was adjusted to 8.5 with 50 mM NaOH, and the concentration was adjusted to 5 mg/mL with lysis buffer. Equal amounts of protein from the control and treatment groups were pooled together as the internal standard. The proteins (50 μg) from the control, the treatment and internal standard were then labeled individually with 400 pmol of Cy3, Cy5 and Cy2, respectively, on ice for 30 min in the dark and then quenched with 1 μL of 10 mM lysine on ice for another 10 min. The Cy3- and Cy5-labeled proteins (50 μg) were combined, and then mixed with 50 μg of Cy2-labeled internal standard. An equal volume of 2 × sample buffer (9 M urea, 2 M thiourea, 4% CHAPS, 130 mM DTT, 1% IPG buffer, pH 3.0–10.0) was then added to the sample, followed by the addition of rehydration buffer (8 M urea, 2% CHAPS, 45 mM DTT, 0.5% IPG buffer, and a trace amount of bromophenol blue, pH 3.0–10.0) to a total volume of 450 μL. Samples were applied to 24-cm, pH 3.0–10.0 IPG strips (Bio-Rad), and isoelectric focusing was performed using the IPGphor IEF system (GE Healthcare). The isoelectric focusing program was set as follows: 50 V for 14 h, Grd 500 V for 30 min, Step 500 V for 1 h, Grd 1000 V for 30 min, Step 1000 V for 1 h, Grd 8000 V for 3 h, and Step 8000 V 30000 Vh. The IPG strips on the concentrator were equilibrated in buffer A (375 mM Tris–HCl [pH 8.8], 6 M urea, 29.3% glycerol, 2% SDS, 1% DTT and a trace amount of bromophenol blue) for 15 min at room temperature and followed by equilibration with buffer B (375 mM Tris–HCl [pH 8.8], 6 M urea, 29.3% glycerol, 2% SDS, 2.5% iodoacetamid and a trace amount of bromophenol blue) for another 15-min incubation at room temperature. Homogeneous polyacrylamide gels (12%) were precast with low fluorescence glass plates using an Ettan DALT six-gel caster, and IPG strips were placed on top of it. Strips were overlaid with 0.5% Agarose-LE (Affymatrix, Santa Clara, CA, USA) in 1 × running buffer containing bromphenol blue and were run for 14–16 h (2 W per gel, overnight) at 16°C in an Ettan DALT six electrophoresis system (GE Healthcare). All electrophoresis procedures were performed in dim light or in the dark. After the run was completed, the 2-D DIGE gels were scanned *in situ* using a Typhoon 9410 Variable Mode Imager (GE Healthcare) according to the manufacturer’s instructions.

All gel images were analyzed by the DeCyder Differential Analysis Software (Version 7.0, GE Healthcare) according to the manufacturer’s recommendation. Briefly, a DeCyder differential in-gel analysis (DIA) module was performed for image analysis between samples within the same gel, while a DeCyder biological variation analysis (BVA) module was performed for pairwise image analysis among multiple gels. Ratios of differentially expressed proteins were shown as fold changes between the pectoral muscle samples of treated and control groups. An increase of protein abundance in the treated group was expressed as a positive value while a negative value denoted a decrease in protein abundance. Protein spots were normalized using the corresponding spot on the pooled internal standard on every gel, and student’s *t*-test on logged ratios were used to compare the average spot volume of all detectable protein pairs. Differential expressions were observed visually by using different colour channels for the treated and control groups.

### Identification of differentially expressed proteins

The protein spots were excised from the gels and destained for 30 min in 100 μL of acetonitrile (50%) and 25 mM NH_4_HCO_3_ (pH 8.0, 50%) until the gel particles were transparent. The particles were then dehydrated for 10 min with acetonitrile (100%), dried for 30 min using a Speed-Vac system, and digested for 1 h at 4°C in 10 μL of trypsin solution (10 ng/μL in 25 mM NH_4_HCO_3_). After the removal of excess trypsin by pipette, the reaction was incubated at 37°C for 12 h, followed by addition of 30 μL of 5% (v/v) TFA (37°C, 1 h), and 30 μL of 50% (v/v) acetonitrile containing 2.5% TFA. After 1 h incubation at 30°C, the supernatants were pooled and dried to 10 μL using a vacuum concentration system.

The digested protein spots were identified by LC-Chip-ESI-QTOF-MS instrument (Q-TOF 6520, Agilent, Santa Clara, CA, USA) equipped with capillary pump (G1382A), nano pump (G2225A), autosampler (G1377D), and chip cube (G4240A). The LC-Chip used (Agilent) consisted of a Zorbax 300SB-C18 enrichment column (40 nL, 5 μm) and a Zorbax 300SB-C18 analytical column (75 μm × 43 mm, 5 μm). The loading flow rate was 4 μL/min, and the loading mobile phase was 0.1% formic acid (solvent A). Elution was performed with a binary mixture of solvents A and B (0.1% formic acid in acetonitrile) as follows: solvent B of 3–8% for 1 min, 8–40% for 5 min, 40–85% for 1 min, and 85% for 1 min. The chip flow rate was 300 nL/min. The MS conditions were: positive ion mode, Vcap of 1900 V, drying gas flow rate of 5 L/min, drying gas temperature of 350°C, fragment voltage of 175 V, skimmer voltage of 65 V, and reference masses m/z of 149.02332 and 1221.02332. The digested samples were diluted in 20 μL of 0.1% formic acid, centrifuged for 5 min at 10,000 × *g*, and 10 μL of the supernatant was injected. The spectra were calibrated using the mass reference standards of purine and HP-0921 (121.050873 and 922.009798, respectively; Agilent). The tandem mass spectra were retrieved using the Mass Hunter software (VersionB.02.01, Agilent). Before the MS/MS data search, a peak-list was generated by the Mascot Distiller software (Version 3.2.1.0, Matrix Science, Boston, MA, USA). The MS/MS data were searched against Mascot 2.2 (Matrix Science) applied to NCBInr (released March 2011; 13,473,798 sequences) with the parameters of carbamidomethyl (C) and oxidation (M) as the fixed and variable modifications, respectively: taxonomy, all entries; enzyme, trypsin/P; missed cleavages, 1; peptide tolerance, ± 20 ppm, and MS/MS tolerance, ± 0.02 Da. When the identified peptides were matched to multiple members of a protein family or a protein appeared under the same name and accession number, the match was made in terms of the higher Mascot score, the putative function and the differential patterns of the protein spots on the 2-D DIGE gels. Protein identifications were accepted if they established a probability greater than 95% and contained at least 2 identified peptides having maximal peptide coverage (Additional file 2: Table S2).

### Bioinformatic analysis

The ClueGo software with the Gene Ontology database (released June 2012) and Kyoto encyclopedia of genes and genomes (KEGG) database (released October 2012) was used to classify identified proteins into specific functional terms and metabolic pathways. The gene ontology analysis based on biological process and enrichment analysis was performed by the right-side hyper-geometric statistic test and its probability value was corrected by Bonferroni’s method [52]. To understand the differential proteins enriched into biological pathway, it is analyzed by ClueGo plug-in of cytoscape software. Symbol ID number of 22 differential proteins was put in the software. Pathway enrichment analysis used the *G. gallus* database from the Kyoto encyclopedia of genes and genomes (KEGG) database by the right-side hyper-geometric statistic test and its probability value was corrected by Bonferroni’s method.

A protein interaction network of the differentially regulated proteins was analyzed using the online database resource Search Tool for the Retrieval of Interacting Genes (STRING 9.1, http://string-db.org/) [53]. The protein regulation networks and protein interaction maps are in the *Gallus gallus* molecular network database. The network nodes are the proteins, and the edges represent the predicted functional associations. An edge may be drawn with up to seven differently coloured lines; these lines represent the existence of the seven types of evidence used in predicting the associations. The interactions between the imported proteins and all proteins stored in the database were then identified.

### Determination of differentially expressed proteins by quantitative real-time PCR (qPCR)

Total RNA extraction and cDNA synthesis of the pectoral muscle of control and treatment groups were performed by using TRNzol-A^+^ and Fast Quant RT Kit (with gDNase) (TIANGEN), according to the manufacturer’s instructions. The quality and concentration of RNA were detected using agarose gel electrophoresis and a spectrophotometer (Ultrospec 2100 pro, GE Healthcare). Seventeen differentially expressed proteins from two major functional groups (Carbohydrate metabolism and energy production, Cytoskeleton) were chosen for qPCR analysis. Specific primers for target genes of the important proteins were designed using the primer BLAST of NCBI and nucleotide information in GenBank (Additional file 3: Table S3). The qPCR was conducted using the iCycler iQ5 system (Bio-Rad). The 20-μL PCR reaction system contained 1 μL of cDNA, 0.5 μL of each primer (10 μM), 10 μL of Super Real PreMix (SYBR Green) (TIANGEN) and 8.2 μL of water. The fold-change of differentially expressed proteins on mRNA level was calculated using the IQ^TM^5 software (Bio-Rad) with the 2 ^−ΔΔCt^ method [54]. All operational program for qPCR strictly followed the MIQE [55].

## Results

The broiler chickens grew normally throughout the experiment [50], as reported before dietary supplementation with *E. faecium* did not significantly promote the growth rate and feed intake of broilers. However, the feed conversion efficiency was improved.

### Carcass traits

**Figure 1:**
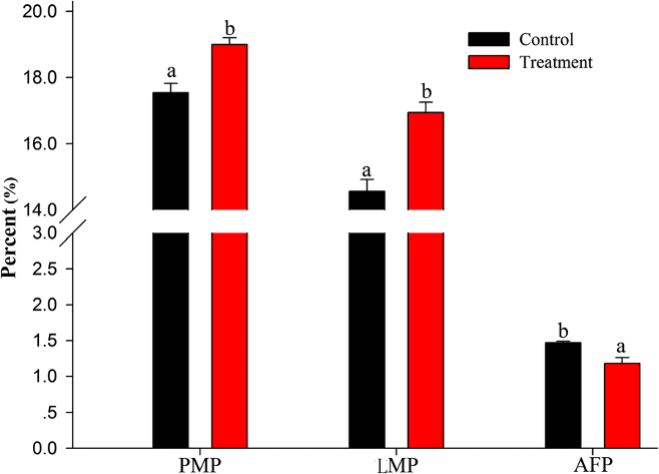
**Effects of dietary** ***E. faecium*** **on the carcass quality of 42-day-old AA broilers.** Different letters represent significant difference at *p* < 0.05. PMP = pectoral muscle weight/body weight, LMP = leg muscle weight (drumstick weight)/body weight, and AFP = abdominal fat weight/body weight.

The weights of pectoral muscle, leg muscle and abdominal fat as a percentage of live body weight are presented in Figure 1. Significant differences were observed between the treatment and control groups on day 42 (*p* < 0.05). Broilers fed *E. faecium* showed improved carcass composition with relatively more pectoral and leg muscles and less abdominal fat.

### Meat characteristics

The effects of *E. faecium* supplementation on meat pH and colour are presented in Table 1. At 45 min and 24 h post mortem, the pH values of the pectoral muscle of the treatment group were higher (*p* < 0.05) than that of the control group. The meat colour didn’t change as much as meat pH value. At 45 min post mortem, only the lightness values showed significant difference (*p* < 0.05) between control and treatment, although the lightness, redness and yellowness values of the pectoral meat of the treatment group were numerically lower than those of the control group. Moreover, the treated broilers had the higher pH values and the darker and less yellow meat (*p* < 0.05). At 24 h post mortem, a significant difference was only observed in the yellowness value. The treated broilers had the higher pH values and the less yellow meat. However, no significant difference was detected in the meat redness and lightness in this study.

**Table 1:** **Effects of the dietary probiotic** ***E. faecium*** **on the pH and colour of pectoral muscles of 42-day-old AA broiler chickens (mean ± S.D.)** ^*^

Index	Control	***E. faecium***	***p*** value
pH at 45 min post mortem	6.15 ± 0.26^a^	6.57 ± 0.17^b^	0.018
pH at 24 h post mortem	5.77 ± 0.10^a^	6.11 ± 0.13^b^	0.008
Meat colour at 45 min post mortem			
Lightness	45.56 ± 0.28^a^	43.61 ± 0.52^b^	0.002
Redness	1.75 ± 0.23^a^	1.47 ± 0.11^a^	0.117
Yellowness	5.08 ± 0.44^a^	4.55 ± 0.10^a^	0.096
Meat colour at 24 h post mortem			
Lightness	49.29 ± 1.29^a^	48.60 ± 0.28^a^	0.248
Redness	1.20 ± 0.07^a^	1.21 ± 0.37^a^	0.959
Yellowness	5.72 ± 0.17^a^	4.18 ± 0.21^b^	0.001

*Data of the same row with different letters (a or b) are significantly different at p<0.05.

**Figure 2:**
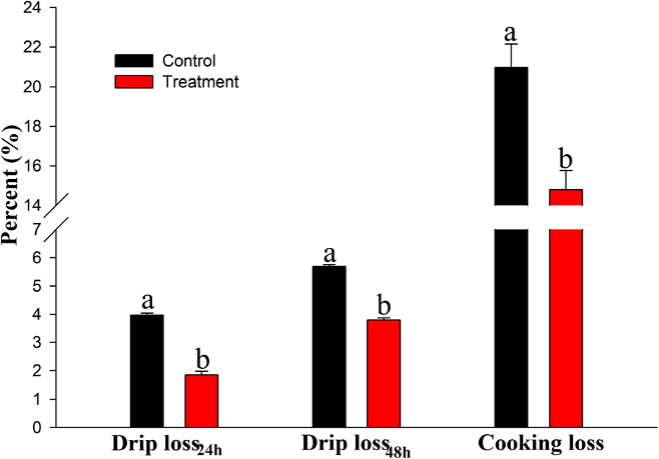
**Effects of dietary** ***E. faecium*** **on the water holding capacity of the pectoral muscles of 42-day-old AA broilers.** Different letters represent significant difference at *p* < 0.05.

The pectoral muscle of broilers fed *E. faecium* had significantly less cooking loss and drip loss than the control birds (Figure 2). Moreover, a positive relationship was found between pH values and the water holding capacity of the muscles.

### Identification and comparison of differentially abundant proteins

**Figure 3:**
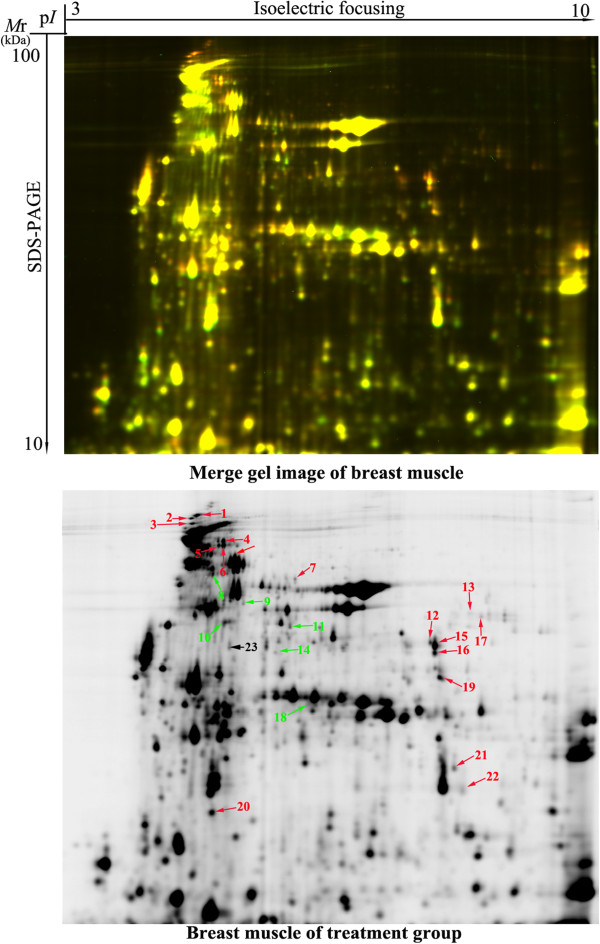
**2-D DIGE profile of the pectoral muscles of 42-day-old AA broilers fed dietary** ***E. faecium*** **.** Protein spots showing significant differences (1.5-fold, *p* < 0.05) were cut out and identified by LC-Chip-ESI-QTOF-MS. Protein spots of differential abundance with known identities are number-labeled in red and green for up-regulation and down-regulation, respectively. Protein spot number-labeled in black is not differential expression between control and treatment groups.

**Figure 4:**
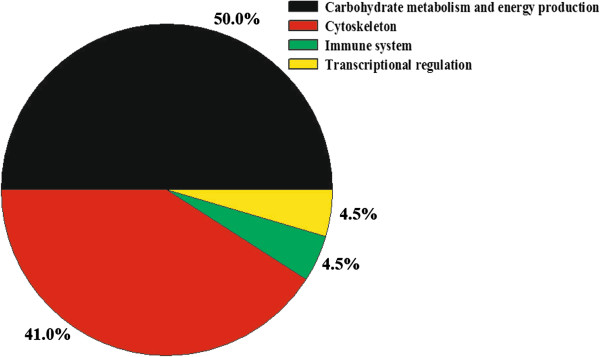
**Functional classification of the proteins of differential abundance identified from the pectoral muscles of 42-day-old AA broilers fed dietary** ***E. faecium*** **.**

A total of 1631 protein spots were detected on 2-D DIGE gels of pectoral muscle. The molecular weights and *p*I values ranged from 10 to 100 kDa and 3.0 to 10.0, respectively (Figure 3). Abundant proteins, especially housekeeping protein β-actin were enriched in random sampling of spots on the gels very well. Moreover, the abundance of β-actin protein (spot 23) was not differentially expressed between control and treatment groups (p > 0.05, Figure 3, Table 2) indicating the reproducibility of the experiment is convincible. The most significant changes (1.5-fold, *p* < 0.05) between control and treatment groups were selected for protein identification by LC-Chip-ESI-QTOF-MS. Except for unidentified proteins due to weak spectra, 22 altered spots were identified (Table 2). These proteins were grouped into four categories based on biological functions: carbohydrate and energy metabolism (50%), cytoskeleton (41%), chaperone protein (4.5%) and transporter (4.5%) (Figure 4). Those related to carbohydrate and energy metabolism and cytoskeleton were predominant and accounted for approximately 90% of the differential abundance proteins identified.

**Table 2:** **Differential proteins classified by biological process in the pectoral muscles of 42-day-old AA broiler chickens fed the dietary probiotic** ***E. faecium*** ^**a**^

Spot no.	Protein name	Accession no.	Symbol ID	***M*** r (kDa)/p ***I***	Sequence coverage (%)	Matched	Mascot score	Av. ratio	***p*** value
**Carbohydrate and energy metabolism**									
7	Phosphoglucomutase-1 (EC = 5.4.2.2)	gi|84619526	PGM1	67.063/8.98	40	49	851	1.7	2.60E − 04
12	L-lactate dehydrogenase A chain (EC = 1.1.1.27)	gi|45384208	LDHA	36.776/7.75	48	30	542	1.7	2.40E − 03
13	Mitochondrial creatine kinase (EC = 2.7.3.2)	gi|268370038	CKMT2	45.851/8.72	28	10	169	2.0	4.20E − 03
14	Pyruvate kinase muscle isozyme (EC = 2.7.1.40)	gi|45382651	PKM2	58.434/7.29	18	22	206	−1.5	1.70E − 04
15	Creatine kinase M-type (EC = 2.7.3.2)	gi|45382875	CKM	43.529/6.50	16	16	75	1.8	1.90E − 04
17	Fructose-1,6-bisphosphatase 2 (EC = 3.1.3.11)	gi|50762391	FBP2	37.364/8.09	35	25	621	1.7	2.60E − 03
18	β-Enolase (EC = 4.2.1.11)	gi|46048765	ENO3	47.566/7.28	21	22	352	−1.5	2.20E − 02
19	Glyceraldehyde-3-phosphate dehydrogenase (EC = 1.2.1.12)	gi|46048961	GAPDH	35.989/8.71	46	38	557	1.6	2.10E − 03
20	Phosphoglycerate kinase (EC = 2.7.2.3)	gi|45384486	PGK	45.087/8.31	10	43	94	1.5	1.80E − 02
21	Phosphoglycerate mutase 1 (EC = 3.1.3.13)	gi|71895985	PGAM1	29.051/7.03	37	9	120	1.7	5.70E − 03
22	Creatine kinase M-type (EC = 2.7.3.2)	gi|45382875	CKM	43.529/6.50	15	5	103	1.9	1.50E − 02
**Cytoskeleton**									
1	Myosin-3	gi|165973976	MYH3	223.88/5.65	19	59	1481	1.9	7.10E − 03
2	Myosin, heavy chain 1	gi|61657934	MYH1	223.746/5.61	12	41	1209	1.8	1.40E − 03
3	Myosin, heavy chain 2, skeletal muscle	gi|45383668	MYH2	223.716/5.71	14	39	1039	2.0	6.80E − 04
5	α-Actinin-2	gi|46048687	ACTN2	104.779/5.26	65	63	472	1.9	3.40E − 04
8	Myosin, heavy chain 6	gi|61657939	MYH6	223.976/5.63	16	62	1288	−1.6	3.30E − 03
9	Myosin, heavy chain 7B, beta	gi|45383005	SSMHC	88.521/5.33	3	8	66	−1.8	1.40E − 03
10	Myosin, heavy chain 15	gi|45382109	MYH15	223.804/5.61	2	13	88	−1.6	1.00E − 02
11	Slow myosin heavy chain 1	gi|363746193	SM1	31.625/5.83	20	43	170	−1.5	4.00E − 04
16	Structural muscle protein titin	gi|363735918	TTN	243.174/6.97	8	25	424	1.8	1.60E − 03
**Chaperone protein**									
4	Heat shock 70 kDa protein	gi|55742654	HSP70	70.098/5.66	23	33	798	1.5	7.70E − 04
**Transporter**									
6	Albumin	gi|45383974	ALB	71.868/5.51	62	59	1481	2.1	2.70E − 05
**Housekeeping protein**									
23	β-actin	gi|45382927	ACTB	8.315/9.25	46	46	213	1.01	1.90E-01

^a^Spot no. corresponds to the number of protein spot in Figure 1. Protein name is given when proteins were identified by LC-Chip ESI-QTOF MS. Accession number is the unique number given to mark the entry of a protein in the database NCBInr. Theoretical molecular weight (*Mr*) and isoelectric point (p*I*) of the identified proteins are retrieved from the protein database of NCBInr. Sequence coverage is the ratio of the number of amino acids in every peptide that matches with the mass spectrum divided by the total number of amino acids in the protein sequence. Matched peptide is the number of paring an experimental fragmentation spectrum to a theoretical segment of protein and searched peptide is the total searched peptide. Mascot score is searched against the database NCBInr. Av. ratio and *p* value are calculated using DeCyder software version 7.0. Av. ratio is the rate of expression abundance of protein in the treatment muscle and the control muscle.

**Figure 5:**
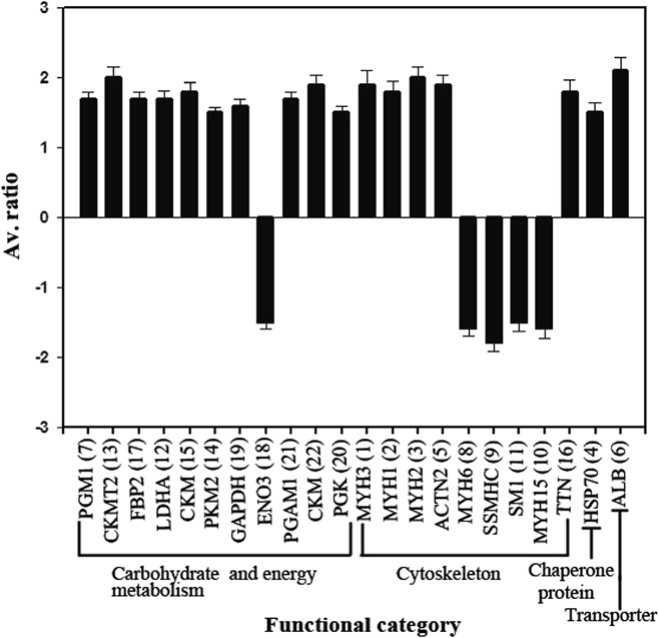
**Quantitative comparisons of the proteins of differential abundance from the pectoral muscles of 42-day-old AA broilers fed dietary** ***E. faecium.*** Av. ratio is the ratio of protein abundance of the treatment compared to the control groups. Positive values indicate the high protein abundance of the treatment group. PGM1, phosphoglucomutase-1; LDHA, L-lactate dehydrogenase A chain; CKMT2, mitochondrial creatine kinase; CKM, creatine kinase M-type; FBP2, fructose-1,6-bisphosphatase 2; GAPDH, glyceraldehyde-3-phosphate dehydrogenase; PGK, phosphoglycerate kinase; PGAM1, phosphoglycerate mutase 1; ENO3, β-enolase; PKM2, pyruvate kinase muscle isozyme; MYH1, myosin, heavy chain 1; MYH2, myosin, heavy chain 2; MYH3, myosin-3; MYH6, myosin, heavy chain 6; MYH15, myosin, heavy chain 15; SSMHC, myosin, heavy chain 7B, beta; ACTN2, α-actinin-2; TTN, structural muscle protein titin; SM1, slow myosin heavy chain 1; HSP70, heat shock 70 kDa protein; ALB, albumin.

A comparison of proteins of differential abundance showed that more protein species were up-regulated in chickens fed *E. faecium* (16 of 22) (Figure 5). Of the 16 up-regulated protein species, nine proteins were involved in carbohydrate metabolism, five in cytoskeleton, one in chaperone protein and one in transporter. The four of six down-regulated proteins in chickens fed *E. faecium* were mainly related to cytoskeleton.

### Bioinformatics analysis of proteins of differential abundance

**Figure 6:**
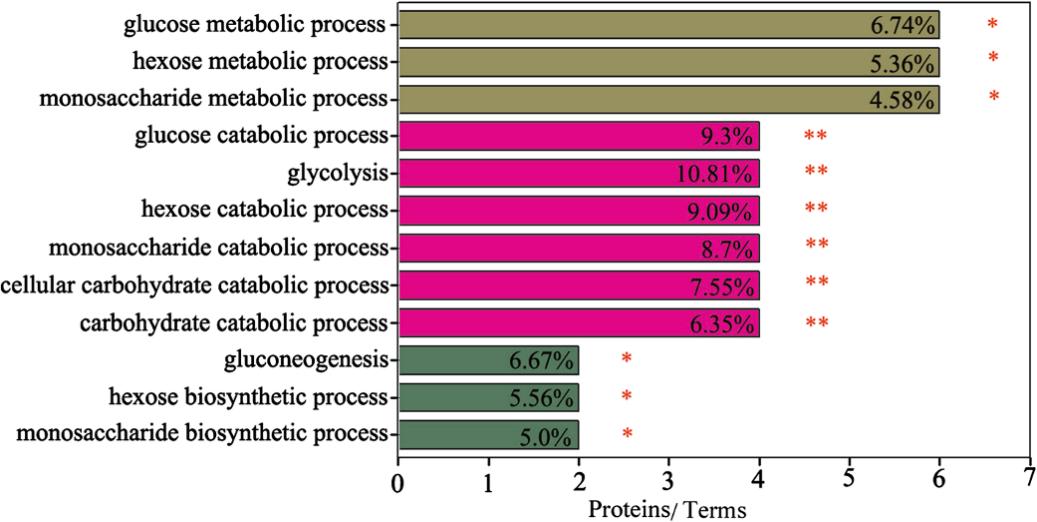
**Functional enrichment analysis of differential abundance of proteins from the pectoral muscles of 42-day-old AA broilers fed dietary** ***E. faecium*** **using the ClueGO software.** * and ** indicate significant enrichment at *p* < 0.05 and *p* < 0.01, respectively.

GO annotation and KEGG pathway enrichment analysis were used to determine the biological characterization and statistical significance of the proteomics data of the pectoral muscle. The ClueGo software identified three highly over represented functional groups (Figure 6), including carbohydrate metabolism, catabolism and anabolism processes. Glucose metabolic processes (metabolism, glycolysis and gluconeogenesis) were the leading terms (a term with statistical significance or with lowest *p* value), respectively. When glucose metabolic process was used as the leading term, PGM1 (spot 7, up-regulated), LDHA (spot 12, up-regulated), PKM2 (spot 14, down-regulated), FBP2 (spot 17, up-regulated), GAPDH (spot 19, up-regulated) and PGK (spot 20, up-regulated) were found to be enriched. When glycolysis process was used as the leading term, LDHA (spot 12, up-regulated), PKM2 (spots 14, down-regulated), GAPDH (spot 19, up-regulated) and PGK (spots 20, up-regulated), were found to be enriched. With gluconeogenesis as the leading term, FBP2 (spot 17, up-regulated) and GAPDH (spot 19, up-regulated) were enriched. This functional enrichment analysis indicated that the *E. faecium* supplement had effects on the anabolism and catabolism of carbohydrate in pectoral muscle of broilers.

The biological pathway is a real functional unit in living systems. Pathway enrichment analysis can help identify key pathways for biological processes. KEGG pathway enrichment analysis of the differential proteins revealed that 14 differentially expressed proteins were significantly enriched in the four pathways (Table 3). These proteins were involved in glycolysis/gluconeogenesis, pentose phosphate pathway, pyruvate metabolism, and tight junction. These results indicated these proteins play the significant role in the improvement of meat quality after feeding *E. faecium* and only very few pathways are induced.

**Figure 7:**
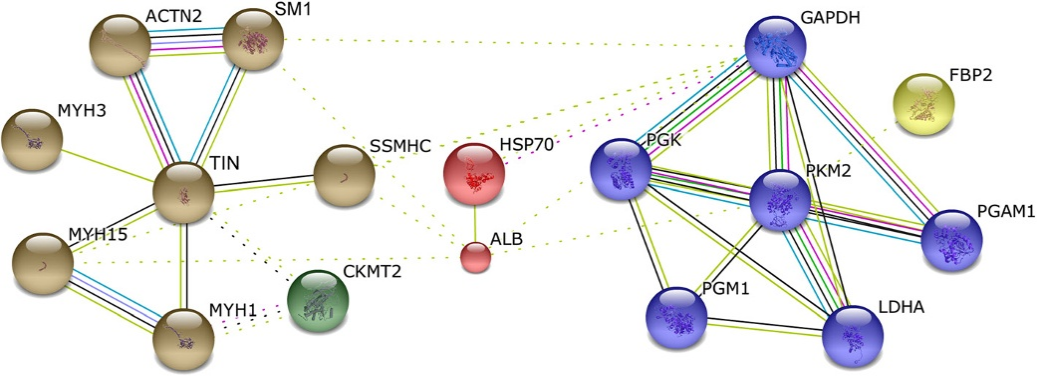
**Biological interaction network of the proteins of differential abundance from the pectoral muscles of 42-day-old AA broilers fed dietary** ***E. faecium.*** Lines between proteins indicate evidence of association. Red indicates fusion, green indicates neighborhood, blue indicates co-occurrence, purple indicates experimental evidence, yellow indicates text-mining evidence, light blue indicates database evidence, and black indicates coexpression. MYH1, myosin, heavy chain 1; MYH3, myosin-3; MYH15, myosin heavy chain 15; SSMHC, myosin, heavy chain 7B, beta; ACTN2, α-actinin-2; TTN, structural muscle protein titin; SM1, slow myosin heavy chain 1; PGM1, phosphoglucomutase-1; LDHA, L-lactate dehydrogenase A chain; CKMT2, mitochondrial creatine kinase; FBP2, fructose-1,6-bisphosphatase 2; GAPDH, glyceraldehyde-3-phosphate dehydrogenase; PGK, phosphoglycerate kinase; PGAM1, phosphoglycerate mutase 1; PKM2, pyruvate kinase muscle isozyme; HSP70, heat shock 70 kDa protein; and ALB, albumin.

Protein networks may provide insights into the biological process involving several proteins. Using the online tools of STRING 9.1, 17 proteins acted as key nodes with various relationships in biological interaction networks (BIN) (Figure 7). Eight proteins (47.1%) were related to carbohydrate and energy metabolism, seven to cytoskeleton, one to chaperone and one to transporter. The results indicate that four main clusters could be individuated in the interaction map and summarized into significant interactions with the other proteins in the map. Of these, PKM2, GAPDH, PGK, LDHA and PGAM1 were the highest degree nodes.

**Table 3:** **Enriched KEGG pathway-based sets of differential proteins in the pectoral muscles of 42-day-old AA broiler chickens fed dietary probiotic** ***E. faecium*** ^**a**^

Pathway name	Count	Proteins	***p*** value	***q*** value
Glycolysis/Gluconeogenesis	7	PGM1 (spot 7), LDHA (spot 12), PKM2 (spot 14), FBP2 (spot 17), GAPDH (spot 19), PGK1 (spot 20), and PGAM1 (spot 21)	5.03E − 10	2.51E − 09
Pentose phosphate pathway	2	PGM1 (spot 7) and FBP2 (spot 17)	3.70E − 03	1.85E − 02
Pyruvate metabolism	2	LDHA (spot 12) and PKM2 (spot 14)	1.07E − 02	5.34E − 02
Tight junction	7	MYH3 (spot 1), MYH1 (spot 2), MYH9 (spot 3), ACTN2 (spot 5), MYH6 (spot 8), MYH7B (spot 9), and MYH15 (spot 10)	2.97E − 07	1.49E − 06

^a^The number of count refers to the amount of proteins involved in the pathway. *p* values are calculated according to a hypergeometric test, *q* values represent *p* values corrected for multiple testing using the false discovery rate method.

### Test gene expression of differential expressed proteins

**Figure 8:**
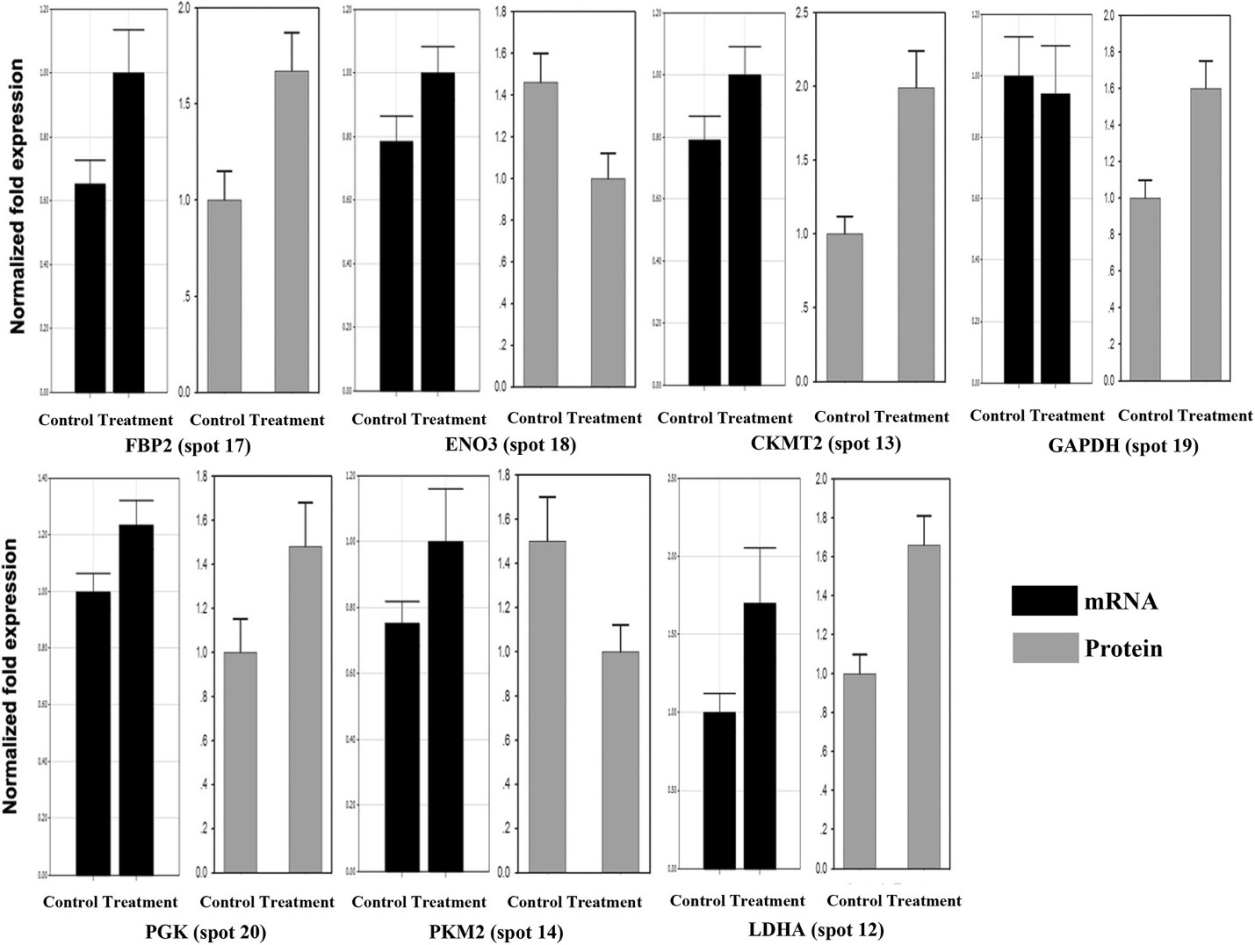
**Validation using qPCR of seven proteins of differential abundance from the pectoral muscles of 42-day-old AA broilers fed dietary** ***E. faecium*** **at the mRNA level.** Samples were normalized with the reference gene 28S rDNA. FBP2, fructose-1,6-bisphosphatase 2; GAPDH, glyceraldehyde-3-phosphate dehydrogenase; ENO3, β-enolase; LDHA, L-lactate dehydrogenase A chain; CKMT2, mitochondrial creatine kinase; PGK, phosphoglycerate kinase; PKM2, pyruvate kinase muscle isozyme.

The correlation between mRNA expression levels and protein abundances depends on several biological factors, such as translation efficiency and protein half-life. Therefore, to manipulate meat quality at gene level, it is important to determine the mRNA expression that regulates meat quality at a protein level. Based on the KEGG pathway and GO analysis of the differentially regulated proteins, seven proteins, i. e. GAPDH, LDHA, PGK1, PKM2, FBP2, CKMT2 and ENO3, were identified as potentially important regulators of meat quality. Further qPCR analysis of these proteins at the mRNA level (Figure 8) demonstrated their roles in glycolysis. The abundance of LDHA, PGK1, FBP2 and CKMT2 were consistent with their mRNA expression levels. However, the mRNA and protein levels of GAPDH, PKM2 and ENO3 were inconsistent. This inconsistency may reflect the unsynchronized abundances of mRNA and proteins.

## Discussion

The ban of antibiotics as growth promoters has increased the search for alternative feed additives for poultry production. Probiotics are one promising alternative due to their positive effects on broiler growth performance, including carcass traits and meat quality [14, 33]. Our data reveals that the probiotic organism *E. faecium* increases the carcass percentage of both pectoral and leg muscles and reduces abdominal fat percent. This result is in agreement with previous results that broiler chickens fed *E. faecium* had significantly less abdominal fat [33, 56]. The meat quality of the pectoral muscle was also improved in terms of pH, colour, and water-holding capacity by feeding the probiotic which triggered the differential expression of 22 proteins. These proteins are involved in carbohydrate metabolism, cytoskeleton, chaperone and transportation. Of the 22 proteins, the 17 linked in the BIN are mainly involved in carbohydrate and energy metabolism and tight junction pathways, suggesting roles in the regulation of meat quality improvement. In combination with our previous studies [50], the main probiotic effects of *E. faecium* occur in the intestine by improving intestinal microbiota composition and mucosa ultrastructure, enhancing nutrient absorption and reducing energy consumption. Our unpublished liver proteome data of broilers fed *E. faecium* identified differentially expressed proteins related to substrate metabolism, and the antioxidant, and immune systems. These differential proteins changed the pentose phosphate and citric acid pathways, enhanced the anabolic metabolism of amino acids and improved antioxidant and immune capacity. As a result, the physio-biochemical indexes of muscle (pH, colour, and drip loss) are improved.

Conversion of muscle to meat is regulated by complex physicochemical processes during post mortem storage [57]. These processes determine subsequent meat quality [58]. In live broilers, aerobic oxidation and glycolysis are important pathways for the provision of energy for muscle contraction and growth. After slaughter, the internal supply of oxygen rapidly diminishes and aerobic respiration ceases after cessation of blood circulation. Anaerobic respiration upregulates immediately, as glycolysis becomes the only pathway in muscle generating energy [59]. Post mortem glycolysis results in an accumulation of lactate and a decline in muscle pH. The reduced pH changes the activity of enzymes. This was reflected in our data (see Table 2) as the expression of a number of enzymes changed in the pectoral muscle after *E. faecium* supplementation as indicated by up-regulation of eight proteins: phosphoglucomutase-1 (PGM1, spot 7), mitochondrial creatine kinase (CKMT2, spot 13), creatine kinase M-type (CKM, spots 15 and 22), fructose-1,6-bisphosphatase (FBP2, spot 17), L-lactate dehydrogenase A chain (LDHA, spot 12), glyceraldehyde-3-phosphate dehydrogenase (GAPDH, spot 19), phosphoglycerate mutase 1 (PGAM1, spot 21), and phosphoglycerate kinase (PGK, spot 20).

Phosphoglucomutase catalyses the conversion of glucose 1-phosphate and glucose 6-phosphate in glycogenesis [60]. Fructose 1,6-bisphosphatase mainly converts fructose 1,6-bisphosphate to fructose 6-phosphate in gluconeogenesis and reductive the pentose phosphate cycle [61, 62]. Therefore, up-regulation of phosphoglucomutase-1 (PGM1, spot 7) and fructose 1,6-bisphosphatase (FBP2, spot 17) in the pectoral muscle indicates improved storage of energy substrates. Moreover, energy production by glycolysis plays a vital role in the contraction and movement of muscles [63]. Significant up-regulation of glycolytic enzymes such as LDHA, PGAM1, CKM, GAPDH and PGK were observed in the pectoral muscle of broilers fed *E. faecium*. CKM is involved in the creatine/phosphocreatine shuttle. Increased expression of CKM suggests that meat tenderness is increased as a result in the delay of post mortem glycolytic activity due to greater reservoirs of phosphocreatine. Higher CKM levels also indicate that ATP stored in muscle cells is depleted slower and sarcomere contraction is delayed. This process also increases meat tenderness [64]. In addition, GAPDH and CKM have been reported to be related to myofibril degradation and produce tender meat [65]. Increases in the expression of GAPDH and CKM also occurred in broiler chickens fed *E. faecium* of our experiment.

The contribution of metabolic enzymes to meat traits is complex [66]. Enolase catalyzes 2-phosphoglycerate to phosphoenolpyruvate during glycolysis [67] and pyruvate kinase catalyzes the transfer of a phosphate group from phosphoenolpyruvate to adenosine diphosphate (ADP) in the aerobic glycolysis pathway, yielding pyruvate and adenosine triphosphate (ATP). Pyruvate will be converted into lactic acid by lactic dehydrogenase. In the present study, down-regulation of β-enolase (ENO3, spot 18) and pyruvate kinase muscle isozyme (PKM2, spot 14) in broilers fed *E. faecium* would reduce the conversion of 2-phosphoglycerate to pyruvic acid. This would reduce the level of lactic acid and increase pH in the pectoral muscle. It has been reported that meat with a higher pH has higher water holding capacity [68]. Thus, the increased pH and water holding capacity of muscle from broilers fed *E. faecium* is likely related to down-regulated expression of β-enolase and pyruvate kinase muscle isozyme.

The second main physiochemical change after slaughter is the transformation of phosphocreatine (PCr) to creatine and hydrolysis of ATP [69]. The up-regulated creatine kinase (CKMT2, spot 13; CKM, spots 15 and 22) in the muscle of broilers fed *E. faecium* enhanced the accumulation of PCr and production of ATP because creatine kinase participates in the interconversion of creatine and ATP to create phosphocreatine (PCr) and ADP [70]. Furthermore, ATP splitting in the muscle *in situ* post mortem can increase the water holding capacity of muscles. This result is consistent with previous findings in Casertana pigs [71]. In the present study, higher CKM levels in the muscle of broilers fed *E. faecium* increased muscle moisture content. The normal functionality of bird muscle are usually associated with myofibrillar structure, such as actin, myosin, cofilin, destrin, titin and tubulin [72]. Degradation of cytoskeletal proteins to smaller peptides weakens the myofibrillar lattice and influences meat quality, especially meat tenderness and water holding capacity [73–77]. Calpains degrade muscle proteins faster at higher tissue pH [78, 79]. Moreover, degradation of key myofibrillar proteins increases water holding capacity [68]. In this study, the higher pH values in the broilers supplemented with *E. faecium* was associated with degradation of structural proteins and improvement in water holding capacity. As the meat pH value was higher than the *p*I values of myofibrillar proteins, this can also increase the binding of water molecules. More light would be absorbed by these water molecules in the muscle, resulting in a darker meat [80]. In our results, the muscle of broilers with *E. faecium* supplementation had higher water-holding capacity and lower lightness and yellowness in colour were in agreement with previous studies [48, 81, 82]. In addition, a negative correlation between lightness of meat colour and pH and a positive correlation between meat redness and pH have been observed [7, 48, 82, 83]. Similar relationships were observed in this experiment. These changes in meat characteristics were the consequences of cytoskeletal protein degradation and pH changes when glycolysis stopped.

Chaperon functions are important for post mortem muscle changes [84]. Chaperon proteins such as HSPs are correlated with meat tenderness [66, 85, 86]. These proteins are involved in folding of newly or denatured proteins [87, 88] and promote the recovery of cell membranes, thus maintaining cell homeostasis [89, 90]. Therefore, the up-regulation of HSP70 (spot 4) in broiler muscle fed *E. faecium* was potentially useful for maintaining muscle cell integrity and repairing denatured proteins, such as desmin and sarcoplasmic proteins. This would be related to the improvement of meat colour and water-holding capacity [91]. It has been reported that desmin, a cytoskeletal protein, is a marker protein for water-holding capacity since higher desmin levels in pig muscle correspond to lower drip loss [92]. Moreover, up-regulation of GAPDH and HSP70 is related to meat tenderness, this result is in agreement with the findings in pigs [71, 93]. Therefore, the enhanced expression of HSP70 (spot 4) and GAPDH (spot 19) also contributes to the improvement of meat tenderness of broilers fed *E. faecium*.

Protein-protein interaction networks provide detailed information of the cellular mechanisms of tissues [94]. The proteins networked in the BIN were mainly involved in glycolysis and tight junction function in the post mortem muscle. This is consistent with that of GO enrichment and KEGG pathway analysis. Only four significantly enriched biological pathways (glycolysis/gluconeogenesis, pentose phosphate pathway, pyruvate metabolism, and tight junction) were observed in this study indicating the central role that they paly in improving meat quality of broilers supplemented with *E. faecium*. Some of the key node proteins that were highly linked in the BIN and enriched in GO term and KEGG pathway analyses were validated at a gene level. Protein abundances of LDHA, PGK1, FBP2 and CKMT2 which were consistent with mRNA levels provide potential targets for genetic manipulation of meat quality.

## Conclusion

*E. faecium* supplementation significantly improved the carcass properties and meat traits of broilers. This was reflected in the differential expression of proteins related to carbohydrate and energy metabolism, cytoskeleton, and molecular chaperones. These proteins are the major regulators of water holding capacity and pH of meat. With dietary *E. faecium* supplementation, expressions of proteins that improve meat quality were enhanced. These new findings increase our understanding of the mechanisms by which feeding probiotics improves chicken meat quality at the level of the proteome.

## Electronic supplementary material

Additional file 1: Table S1: The composition of broiler chicken starter and grower diets (g/kg). (DOC 61 KB)

Additional file 2: Table S2: Peptides identified from pectoral muscles of AA broiler chickens based on Mascot scores. (DOC 82 KB)

Additional file 3: Table S3: The primer sequences used for qPCR analysis of the differentially expressed proteins of the pectoral muscles of AA broiler chickens. (DOC 35 KB)
