# Supplementary material for: Proteome changes underpin improved meat quality and yield of chickens (Gallus gallus) fed the probiotic Enterococcus faecium
Source: BMC Genomics. 2014 Dec 23;15(1):1167. doi: 10.1186/1471-2164-15-1167 (PMC4325948; doi:10.1186/1471-2164-15-1167)
Supplement: Supplementary file 1 — Additional file 1: Table S1: The composition of broiler chicken starter and grower diets (g/kg). (DOC 61 KB) [file 12864_2014_6990_MOESM1_ESM.doc]

**Additional file 1**

**Table S1- The composition of broiler chickens starter and grower diets (g/kg)^a^**.

|  | **Starter diet** | **Grower diet** |
| --- | --- | --- |
| **Ingredients** | | |
| Corn | 548.9 | 593.1 |
| Soybean meal | 306.3 | 246.3 |
| Cottonseed meal | 30.0 | 40.0 |
| Rapeseed Meal | 30.0 | 40.0 |
| Oil | 34.5 | 35.9 |
| Limestone | 16.5 | 14.1 |
| Dicalcium phosphate | 18.3 | 15.9 |
| Salt | 3.0 | 3.0 |
| Methionine | 1.2 | 1.1 |
| Lysine | 1.4 | 0.5 |
| Vitamin/mineral premix^a^ | 10.0 | 10.0 |
| **Calculated nutritive value** | | |
| Metabolizable energy (Mcal/kg) | 2.90 | 2.95 |
| Crude protein | 210.0 | 195.0 |
| Crude fat | 60.3 | 62.4 |
| Calcium | 11.5 | 10.0 |
| Total phosphorus | 6.7 | 6.3 |
| Available phosphorus | 4.7 | 4.3 |
| Lysine | 11.0 | 9.2 |
| Methionine | 4.5 | 4.2 |
| Methionine + Cystine | 8.2 | 7.7 |
| Threonine | 8.4 | 7.7 |
| Tryptophane | 2.8 | 2.5 |
| Arginine | 13.8 | 12.7 |

^a^ Provided the following nutrients (per kg of air-dried diet): Methionine 1.5 g, Vitamin A 10,000 IU, Vitamin D_3_ 3000 IU, Vitamin E 20 mg, Vitamin K 32 mg, Vitamin B_1_ 2.0 mg, Vitamin B_2_ 8 mg, Vitamin B_6_ 3 mg, Vitamin B_12_ 0.06 mg, panthothenic acid 20 mg, niacin 50 mg, folic acid 1.0 mg, biotin 0.15 mg, choline 500 mg, Cu 8 mg, Zn 60 mg, Fe 80 mg, Se 0.15 mg, and I 0.22 mg.
