# Supplementary material for: Proteome changes underpin improved meat quality and yield of chickens (Gallus gallus) fed the probiotic Enterococcus faecium
Source: BMC Genomics. 2014 Dec 23;15(1):1167. doi: 10.1186/1471-2164-15-1167 (PMC4325948; doi:10.1186/1471-2164-15-1167)
Supplement: Supplementary file 2 — Additional file 2: Table S2: Peptides identified from pectoral muscles of AA broiler chickens based on Mascot scores. (DOC 82 KB) [file 12864_2014_6990_MOESM2_ESM.doc]

**Additional file 2**

**Table S2-Peptides identified based on Mascot scores ^a^.**

| **Spot no.** | **Protein name** | **Species** | **Mascot score** | **Peptides** |
| --- | --- | --- | --- | --- |
| 1 | Myosin-3 | *G. gallus* | 1481 | STHPHFVR  IKPLLK  TKIQLEAK  AEDEEEINAELTAK  KLEDECSELKK  NLTEEMAALDETIVK  LEQQVDDLEGSLEQEK  KDFEISQIQSKIEDEQALGMQLQKKIKELQARIEELEEEIEAERTSR  HRADLSRELEEISER  EAEFQKMRRDLEEATLQHEATAAALRKKHADSTAELGEQIDNLQR  MEIDDLASNMESVSKAKANLEK  TLEDQLSEIKTK  MINDLNTQR  HLEEEIKAKNALAHALQSAR  EQYEEEQEAKGELQRALSKANSEVAQWRTKYETDAIQR  LQDAEEHVEAVNAKCASLEKTK  NFDKILAEWK  IAEKDEEIDQLK  MEGDLNEMEIQLSHANR  DTQIHLDDALR  EQVAMVER |
| 2 | Myosin, heavy chain 1 | *G. gallus* | 1209 | LYDQHLGK  IKPLLK  AEDEEEINAELTAK  KLEDECSELK  KDFEISQIQSKIEDEQALGMQLQKKIKELQARIEELEEEIEAERTSR  HRADLSRELEEISER  DLEEATLQHEATAAALR  HADSTAELGEQIDNLQR  MEIDDLASNMESVSK  HLEEEIKAK  ALSKANSEVAQWR  LQDAEEHVEAVNAK  ANSACAALDKK  QKYEETQTELEASQK  IAEKDEEIDQLK  IVESMQSTLDAEIR  EQVAMVER |
| 3 | Myosin, heavy chain 2, skeletal muscle | *G. gallus* | 1039 | NALAHALQASR  ANSACAALDKK  ILAEWK |
| 4 | Heat shock 70 kDa protein | *G. gallus* | 798 | VEIIANDQGNRTTPSYVAFTDTERLIGDAAK  KYDDPTVQSDMK  MKEIAEAYLGK  IINEPTAAAIAYGLDKK  STAGDTHLGGEDFDNR  LRTACER  ARFEELNADLFR  LLQDFFNGKELNK  AMTKDNNLLGK  ITITNDKGR  MVQEAEKYK |
| 5 | α-Actinin-2 | *G. gallus* | 472 | VLAVNQENER  KLEDFR  CQLEINFNTLQTK  ISNRPAFMPSEGKMVSDIAGAWQR  DYESASLTEVR  ATLPEADGERQAILSIQNEVEK  ISASNPYSTVTVEEIR  DQSLQEELAR  QFAAQANVIGPWIQTK  QYEQNIINYK  TINEVETQILTR  GITQEQMNDFRASFNHFDR |
| 6 | Albumin | *G. gallus* | 1481 | DAEHKSEIAHRYNDLKEETFKAVAMITFAQYLQR  LVKDVVDLAQK  LRDSYGAMADCCSKADPERNECFLSFKVSQPDFVQPYQRPASDVICQEYQDNRVSFLGHFIYSVAR  ESDVGACLDTKEIVMR  QQYFCGILK  QLIYLSQKYPKAPFSEVSKFVHDSIGVHK  MMSNLCSQQDVFSGK  DCCEKPIVERSQCIMEAEFDEKPADLPSLVEKYIEDKEVCK  IAKGYESLLEK  TDNPAECYANAQEQLNQHIK  TNCDLLHDHGEADFLK  MPQVPTDLLLETGKKMTTIGTK  RPCFTAMGVDTKYVPPPFNPDMFSFDEKLCSAPAEEREVGQMKLLINLIKRKPQMTEEQIKTIADGFTAMVDKCCKQSDINTCFGEEGANLIVQSR |
| 7 | Phosphoglucomutase-1 (EC=5.4.2.2) | *G. gallus* | 851 | VHIETVK  AYADQKPGTSGLR  QEATLVVGGDGR  IAAANGIGRLVIGQNGILSTPAVSCIIR  FNTANGGPAPEGITDK  KIEEYAICPDLKVDLGTIGKQQFDLENK  IDAMHGVVGPYVK  TGEYDFGAAFDGDGDRNMILGK  SMPTSGALDR  IALYETPTGWKFFGNLMDANKLSLCGEESFGTGSDHIR  NFFTRYDYEEVDADAAGKMMKDLETVMFDR  QLSAGDKVYTVEKADNFEYNDPVDGSVSR |
| 8 | Myosin, heavy chain 6 | *G. gallus* | 1288 | IKPLLK  NLTEEMAVLDETIAK  LRMDLER  KDFEISQIQSKIEDEQALGMQLQKKIKELQARIEELEEEIEAER  ELEEISERLEEAGGATAAQIEMNK  DLEEATLQHEATAAALRK  HADSTAELGEQIDNLQR  MEIDDLASNMESVSK  TLEDQLSEIK  MINDLNTQRARLQTETGEYSRQAEEKDALISQLSR  QGFTQQIEELK  HLEEEIKAKNALAHALQSARHDCELLREQYEEEQEAKGELQR  ANSEVAQWRTKYETDAIQR  LQDAEEHVEAVNAK  LQNEVEDLMVDVERSNAACAALDKK  NFDKILAEWKQKYEETQTELEASQK |
| 9 | Myosin, heavy chain 7B, beta | *G. gallus* | 66 | IQLELNQIK  KMEGDLNEMEIQLSHANR  AITDAAMMAEELKKEQDTSAHLER  LDEAEQIALKGGK  QIQKLEAR  ELSYQTEEDRK |
| 10 | Myosin, heavy chain 15 | *G. gallus* | 88 | MINDLNTQR  LQTETGEYSR  ANSEVAQWR  LQDAEEHVEAVNAK |
| 11 | Slow myosin heavy chain 1 | *G. gallus* | 170 | AITDAAMMAEELKKEQDTSAHLER  LDEAEQLALKGGK  QLQKLEAR  VKELSYQTEEDR |
| 12 | L-Lactate dehydrogenase A chain (EC=1.1.1.27) | *G. gallus* | 542 | DLADELTLVDVVEDK  GEMLDLQHGSLFLK  DYSVTAHSKLVIVTAGAR  FIIPNVVK  ISGFPKHRVIGSGCNLDSARFRHLMGER  ALHPDMGTDADKEHWK  RVHPISTAVKGMHGIKDDVFLSVPCVLGSSGITDVVKMILKPDEEEK  KSADTLWGIQKELQF |
| 13 | Mitochondrial creatine kinase (EC=2.7.3.2) | *G. gallus* | 169 | HNGYDPRTMKHHTDLDASKITHGQFDER  GLSLPPACSR  YYSLTNMSER  AGVHVKLPR  GTGGVDTAAVADVYDISNLDR |
| 14 | Pyruvate kinase muscle isozyme (EC=2.7.1.40) | *G. gallus* | 206 | LDIDSEPTIARNTGIICTIGPASR  LNFSHGTHEYHEGTIK  EATESFASDPITYRPVAIALDTK  GSGTAEVELKK  VTLDNAFMENCDENVLWVDY K  IYVDDGLISLLVK  GKDFVMTEVENGG  MLGSKKGVNLPGAAVDLPAVSEKDIQDLKF  GVEQNVDMVFASFIRKAADVHAVRK  IISKIENHEGVRRFDEIMEASDGIMVARGDLGIEIPAEKVFLAQK  AGKPIICATQMLESMIK  AEGSDVANAVLDGADCIMLSGETAKGDYPLEAVRMQHAIAR |
| 15 | Creatine kinase M-type (EC=2.7.3.2) | *G. gallus* | 75 | PFSSTHNKHK  FSAEEEFPDLSKHNNHMAKVLTPELYKR  HGGYKPTDK  SIKGYSLPPHCSR  GGVHVKLPK |
| 16 | Structural muscle protein titin | *G. gallus* | 424 | YGQEQWEEGDLYDKEK  LKQFGPAHFECR  MINEFGYCSLDYGVAYSRDSGVITCR  YGTDHTSATLIVKDEKSLVEESQLPEGR  MAHEGALPAVAVDQKEKQKPELVLVPEPARVLEGETARFRCRVTGYPLPKVNWYLNSQLIR  YDGIHYLDIVDCKSYDTGEVKVTAENPEGFIEHKVKLEIQQR |
| 17 | Fructose-1,6-bisphosphatase 2 (EC=3.1.3.11) | *G. gallus* | 621 | TPFETDMLTLTRFVMEKGR  ETDDEPSEKDALQPGRK  GKIYSLNEGYAKYFDPAMTEYLKKKKFPEDGSSPYGARYVGSMVADVHRTLMYGGIFMYPANQK  VPLILGSPDDVHEYLACVQK |
| 18 | β-Enolase (EC=4.2.1.11) | *G. gallus* | 352 | AAIAQAGYTDKVVIGMDVAASEFCR  VNQIGSVTESIQACKLAQSHGWGVMVSHRSGETEDTFIADLVVGLCTGQIK  LAKYNQLMRIEEALGDKAK |
| 19 | Glyceraldehyde-3-phosphate dehydrogenase (EC=1.2.1.12) | *G. gallus* | 557 | YDSTHGHFKGTVK  WADAGAEYVVESTGVFTTMEKAGAHLKGGAK  VIISAPSADAPMFVMGVNHEKYDK  IVSNASCTTNCLAPLAK  GAAQNIIPASTGAAK  VIPELNGKLTGMAFRVPTPNVSVVDLTCR  LVSWYDNEFGYSNRVVDLMVHMASKE |
| 20 | Phosphoglycerate kinase (EC=2.7.2.3) | *G. gallus* | 94 | **IVKDLMAK**  **KFVEVVGR**  ALMDKVVEVTGKGCITII  GGGDTATCCAK |
| 21 | Phosphoglycerate mutase 1 (EC=3.1.3.13) | *G. gallus* | 120 | **HGESAWNLENRFCGWYDADL**  **SPAGQQEAR**  **DAGYEFDICFTSVQKR**  **HYGALTGLNKAETAAKHGEAQVK**  **SFDIPPPPMQSDHPFFSTISK**  **YADLTEDQLPTCESLKDTI**  **ARALPFWNEEIVPQIK**  **RVLIAAHGNSLR**  **KAMEAVAAQGK** |
| 22 | Creatine kinase M-type (EC=2.7.3.2) | *G. gallus* | 103 | **HGGYKPTDK**  **TDLNHENLKGGDDLDPK**  **SIKGYSLPPHCSR**  **IEEIFKK**  **FEEILHR** |
| 23 | β-actin | *G. gallus* | 213 | TTMYPGIADR  MQKEITALAPSTMK  EITALAPSTMK  IKIIAPPER  IIAPPER |

^a^ Spot no. corresponds to the number of protein spots in Fig. 1. Mascot scores are derived from ion scores as a non-probabilistic basis for ranking protein hits.
