# Supplementary material for: Proteome changes underpin improved meat quality and yield of chickens (Gallus gallus) fed the probiotic Enterococcus faecium
Source: BMC Genomics. 2014 Dec 23;15(1):1167. doi: 10.1186/1471-2164-15-1167 (PMC4325948; doi:10.1186/1471-2164-15-1167)
Supplement: Supplementary file 3 — Additional file 3: Table S3: The primer sequences used for qPCR analysis of the differentially expressed proteins of the pectoral muscles of AA broiler chickens. (DOC 35 KB) [file 12864_2014_6990_MOESM3_ESM.doc]

**Additional file 3**

**Table S3-The primer sequences used for qPCR analysis of the differentially expressed proteins of the pectoral muscles of AA broiler chickens.**

| **Spot no.** | **Symbol ID** | **Accession no.** | **Gene name** | **Primer sequence (5′→3′)** | **Product size (bp)** | **Tm (°C)** |
| --- | --- | --- | --- | --- | --- | --- |
| 12 | LDHA | gi|45384208 | L-Lactate dehydrogenase A chain | CAAGATCAGCGTGGTTGGTG  CAGCTTGTCCTCCACAACATC | 112 | 62 |
| 13 | CKMT2 | gi|268370038 | Mitochondrial creatine kinase | ACCTGACGCCAGCCATCTAC  GTCTCCTCATCACCAGCCAC | 131 | 62 |
| 14 | PKM2 | gi|45382651 | Pyruvate kinase muscle isozyme | GCCTTCATGGAGAACTGCGA  CATGCCACCGTTCTCAACCT | 168 | 62 |
| 17 | FBP2 | gi|50762391 | Fructose-1,6-bisphosphatase | CAGCTGCTCAACTCCATGCT  CAGTGCCTGCTATGCCGAAC | 94 | 62 |
| 18 | ENO3 | gi|46048765 | β-Enolase | GCACACTTCCTACTGCACCT  TGTGCGTACAACAGGAATG | 125 | 62 |
| 19 | GAPDH | gi|46048961 | Glyceraldehyde-3-phosphate dehydrogenase | GACAGCCATTCCTCCACCTT  GGACCATCAAGTCCACAACAC | 126 | 62 |
| 20 | PGK | gi|45384486 | Phosphoglycerate kinase | GCGGTAACCATGTCTCTCTCC  GGACAGCTGCCTTGATTCTT | 142 | 62 |
| Ref | 28S | FM165415.2 | 28S rRNA | AGGTGCAGATCTTGGTGGTAG  CGCTTAGGACCAACTGACC | 112 | 62 |
